# Sir James Barr and the Medical Profession

**Published:** 1918-10-05

**Authors:** 


### The Hospital

The Workers' Newspaper of Administrative Medicine and Institutional Life, Administration, National Insurance and Health.

No. 1687, Vol. LXV. SATURDAY, OCTOBER 5, 1918. PRICE TWOPENCE

### SIR JAMES BARR AND THE MEDICAL PROFESSION.

The *British Medical Journal* publishes a long article by Sir James Barr entitled "The Future of the Medical Profession." Almost any other title would have suited it equally well, for it has little to say on the future of the medical profession and a great deal to say on a great many other subjects; but there is no reason to complain of this. Sir James Barr is always interesting, always suggestive, and always vigorous. He follows no one, but thinks things out for himself—a rare faculty and a most valuable one—and what he has to say is expressed in good, sound, virile English that it is a pleasure to read. The subject of his article is not so much the future of the medical profession as the future of the human race in this country, and he looks upon the prospect as gloomy. The physically and mentally fit are carrying on the war, while the derelicts are carrying on the race. The honest, hardworking men have to support not only their own families, but also those of knaves, fools, paupers, and wastrels of every description; and what will happen to this country when a moiety of the adult population will be Government officials supported with their wives and families by the other half, who will also have to bear the expenses of the war?

These are very pertinent observations, too much overlooked, and there are many of equal value scattered through Sir James Barr's address. He quotes with approval Dr. D. W. Hunter's dictum that "If to-morrow the tubercle bacillus were non-existent, it would be nothing short of a national calamity." He might have said the same of alcohol. Nature has been engaged for hundreds and thousands of years in breeding, with the aid of natural selection, a race of men who shall be immune to the attacks of the tubercle bacillus. By means of the bacillus she has steadily eliminated the weak, both in body and mind, the idiot, the imbecile, and the weakling. She is halfway through her task, and now we set ourselves to interfere and counteract her beneficent operation. "When on e you interfere with the order of Nature," said Huxley, "there is no knowing where the results will end." By preserving those who are obnoxious to the attack of the tubercle bacillus, and enabling them to breed, we are producing deterioration in the race. It is the same with alcohol. As Dr. Archdall Reid has shown, Nature, through the operation of alcohol itself, has been breeding for thousands of years a race that shall be free from the proclivity to take alcohol in excess; and she is in a fair way to succeed. Those races of men are most sober that have longest had free access to alcohol, for those who indulged most freely died early and left fewer offspring, while those who had least appetite for it survived longer and left more numerous descendants, who inherited the indifference to alcohol of their parents. Those States that enact laws against alcohol and prohibit its use interfere, as far as their prohibition is successful, with this beneficent process, leave the race vulnerable to the influence of alcohol, and produce a reversion to the state of things we witness in some savage and barbarous tribes who have never had access to it, and who are rapidly killed off when ardent spirits are introduced among them. Prohibition of alcohol, if it is really effectual, will secure a sobriety that will, it is true, be universal, but that will endure only as long as the prohibition is effectual. The sobriety will be due to external coercion, not to internal distaste for the drug, and when access to it is once more gained, as it is sure to be sooner or later, the consequences will be disastrous. When once you interfere with the operations of Nature, you never know where the results will end. The frontal attack is always more costly than the out-flanking attack.

At length, after some seven columns of racy disquisition *de omnibus rebus et quibusdam aliis*, Sir James Barr devotes a short paragraph to the future of the medical profession. His opinion is that it will be, or ought to be, the function of the medical practitioner not so much to cure disease as to prevent it; to prevent it, not by the wholesale methods of the medical officer of health, but individually. The task of the general practitioner of the future will be to keep his patients in good health, not to let them become diseased and then try to set them right; usually an impossible task. Sir James

Barr might have pointed out that this revolution has already taken place in one department of medicine. We do not now wait until our teeth are decayed before we visit the dentist. We visit him every six months or so in order that he may detect the earliest departures from health and keep our teeth in repair. Moreover, the ideal that Sir James Barr sets before us has already been achieved by the Chinese, who once attained a grade of civilisation in many respects superior to our own. The custom of the Chinese is, or was, to pay their physicians only as long as these physicians kept them in good health. When illness attacked the patient, the pay of the physicians ceased until health was restored. What has Sir James Barr to say to this arrangement?

